# Supplementary material for: Impact of School and Peer Connectedness on Adolescent Mental Health and Well-Being Outcomes during the COVID-19 Pandemic: A Longitudinal Panel Survey
Source: Int J Environ Res Public Health. 2022 Jun 1;19(11):6768. doi: 10.3390/ijerph19116768 (PMC9180617; doi:10.3390/ijerph19116768)
Supplement: Supplementary file 1 [file ijerph-19-06768-s001.zip › ijerph-1693245-supplementary.pdf]

Supplementary Materials 1: School Recruitment and Comparison

All non-fee-paying secondary schools across four local authorities in South West England, UK (N = 76) were invited to participate in the study through emails to headteachers and pastoral leads. Of these, 30 responded and 19 schools went on to take part in the study at time 1 (pre-COVID). Recruitment stopped once 19 schools had signed up to the study based on sample size calculations designed to detect an effect size of 0.2. Compared to non-participating schools in the target areas, participating schools were more likely to be non-religious and in urban areas but were comparable in terms of eligibility for free school meals and academy status.

Table S1. Comparison of participating and non-participating schools in the target areas (https://www.compare-school-performance.service.gov.uk/; pupil population data available for 2018/2019).

	**Participating Schools, Time 1, 2 & 3 (*n* = 17)**	**Non-Participating Schools in Target Area (*n* = 59)**
**Rural**	6%	19%
**Urban**	94%	81%
**Mixed gender**	94%	93%
**Mean % pupils eligible for free school meals (*during the last 6 years*)**	23.1%	23.4%
**Academy status [1]**	85%	88%
**No religious denomination**	74%	88%

[1] Academies are state-funded schools run by not-for-profit trusts. They are not required to follow the national curriculum but are subject to the same admissions and examinations processes as other state funded schools.

All Year 9 students (aged 13–14 years) were invited to complete an online survey during lesson time in October and November 2019 and again 6 months later during lockdown. Baseline mean response rate across schools was 76.6% (SD = 15.1%), with a total sample size of 2549 at time 1 (T1). At time 2, mean response rate across schools was 33.5%. One thousand and thirty-nine students took part in the time 2 survey, and complete HADS data at both time points were available for 798 (anxiety) and 792 (depression). For the second survey (during lockdown), two schools were unable to participate, so seventeen schools participated at both time points.
